# Development and Evaluation of an Evidence‐Based Management Indicator System for Transitional Care in Preterm Infants Using a Delphi and AHP Approach

**DOI:** 10.1155/jonm/2638144

**Published:** 2026-05-18

**Authors:** Qing Meng, Fei Wang, Kefan Chen, Yingxin Li, Yuan Li, Yanling Hu

**Affiliations:** ^1^ Department of Neonatology Nursing, West China Second University Hospital, Sichuan University, Chengdu, 610041, Sichuan, China, scu.edu.cn; ^2^ Key Laboratory of Birth Defects and Related Diseases of Women and Children (Sichuan University), Ministry of Education, Chengdu, 610041, Sichuan, China, scu.edu.cn; ^3^ West China School of Medicine/West China Hospital, Sichuan University, Chengdu, 610041, Sichuan, China, scu.edu.cn; ^4^ The First Affiliated Hospital, Sun Yat-Sen University, Guangzhou, 510080, Guangdong, China, sysu.edu.cn

**Keywords:** Delphi technique, patient discharge, preterm infant, quality indicators, transitional care

## Abstract

**Background:**

The transition from hospital to home represents a critical and often perilous period for preterm infants. Inadequate discharge preparation frequently leads to adverse outcomes and preventable readmissions.

**Objective:**

This study aimed to develop and evaluate a robust management indicator system to standardize and enhance the quality of transitional care for this vulnerable population.

**Methods:**

A sequential multiphase study was conducted, beginning with a systematic literature review to establish a preliminary indicator pool. A two‐round Delphi survey involving 21 neonatal care experts was then used to refine the indicators and achieve consensus. Finally, the analytic hierarchy process (AHP) was employed to determine the relative weight of each indicator.

**Results:**

The response rate for both Delphi rounds was 100%. The expert consensus reliability (Cr) was 0.967 and 0.964 for the two rounds, respectively, with consensus scores (Cs) of 0.971 for both. Kendall’s W coefficients were 0.385 and 0.187 (both *p* < 0.05), indicating statistically significant agreement among experts across both rounds. The finalized management indicator system comprises 3 equally weighted primary indicators (0.333 each), 17 secondary indicators, and 42 tertiary indicators. Notably, at the secondary level, “Transitional care readiness” (0.234) received the highest weight. Among the tertiary indicators, the highest weight was assigned to “Emergency preparedness skills” (0.162), followed by “Medication administration guidance” (0.054).

**Conclusion:**

The management indicator system developed in this study demonstrates methodological rigor and expert consensus, offering a standardized, weighted framework for assessing transitional care readiness, guiding transitional care processes, and informing evidence‐based management practices for preterm infants. The system’s emphasis on emergency preparedness and family capability assessment highlights areas that may warrant greater attention in current discharge planning approaches, pending further empirical validation.

## 1. Introduction

Preterm birth constitutes a major public health challenge, affecting approximately 10% of all newborns worldwide [1, 2]. These infants, characterized by systemic immaturity and diminished physiological adaptability [3], face a heightened risk of mortality and a spectrum of long‐term morbidities, including significant neurodevelopmental impairments [4–7]. Due to their inherent vulnerability, the majority of these infants require prolonged hospitalization in a highly specialized environment, the neonatal intensive care unit (NICU) [8]. Consequently, the developmental trajectory of this vulnerable population is profoundly shaped by the quality of in‐hospital medical care, the postdischarge home environment, and the proficiency of parental caregiving [9]. The transition from the controlled setting of the NICU to home thus emerges as a period of significant risk [10] that demands systematic attention and concerted support.

Inadequate preparation for this transition is a leading cause of early hospital readmissions, often due to preventable conditions such as respiratory infections and gastrointestinal issues [11]. Such events not only compromise the infant’s immediate health [12] and long‐term development [13] but also impose considerable emotional and financial burdens on families [14]. This challenge is particularly acute in healthcare systems where practices like mother–infant separation [15] in the NICU are common, as this can disrupt the continuity of care and impede parental readiness for the complexities of home care [16]. For instance, studies in China have reported readmission rates as high as 49.06% within the first week of discharge, largely attributed to deficiencies in discharge preparation, transitional support, and subsequent home‐based care [17].

In response to these challenges, the use of quality indicators has become a cornerstone of modern healthcare management. Quality indicators are explicit, measurable elements of practice that can be used to assess the quality of care and track changes over time [18]. The establishment of a scientifically grounded indicator system provides a standardized framework to translate complex clinical guidelines into actionable and assessable components. This process helps to reduce variability in care, identify gaps in service delivery, and guide targeted quality improvement initiatives. For a complex process like the hospital‐to‐home transition of preterm infants, such an indicator system is essential for ensuring that all critical facets of care, from clinical stability to family education, are systematically addressed and evaluated. Moreover, a well‐designed indicator system enables healthcare teams to prioritize interventions based on evidence‐derived weights, ensuring optimal resource allocation during this critical transition period.

To address this gap, it is essential to establish a clear conceptual distinction between the specific transition point and the ongoing management process. In this study, transitional care is conceptualized as a comprehensive continuum of interventions spanning from discharge preparation to postdischarge follow‐up, designed to bridge the gap between hospital‐based medical care and home‐based family care [19]. While discharge represents the formal transition point where infants are released from the hospital [10], it serves as a critical milestone embedded within this broader longitudinal framework of transitional care. Currently, however, quality improvement efforts remain largely confined to standardizing in‐hospital nursing procedures [20, 21], leaving the structured transition to home underdeveloped [22–25]. Therefore, this study was designed to develop and evaluate a comprehensive, multilevel management indicator system that integrates systematic literature evidence with expert consensus to provide healthcare teams with a standardized, weighted framework for optimizing these critical transitions.

## 2. Methods

### 2.1. Study design

**FIGURE 1:**
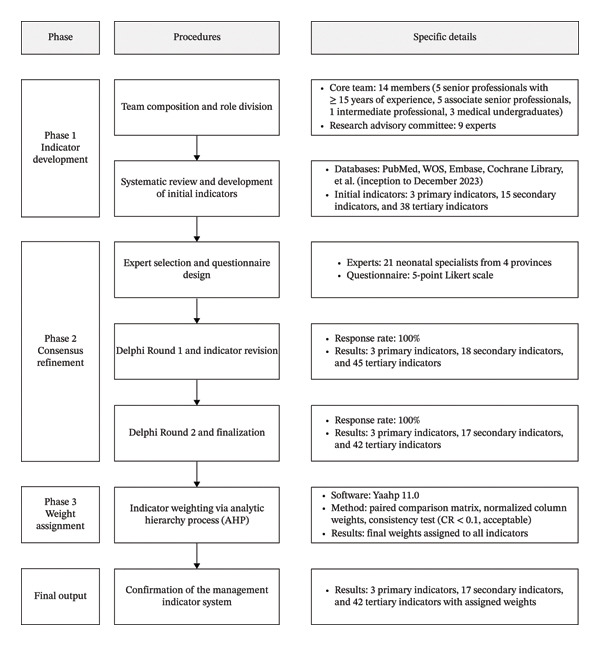
Flowchart of the multiphase study design.

A sequential multiphase research design was employed. The study proceeded in three main phases, as illustrated in Figure 1. The first phase was the development of an initial indicator pool through a systematic literature review. This was followed by the refinement of and consensus on the indicators using a Delphi survey methodology. The final phase involved the assignment of weights to the finalized indicators using the analytic hierarchy process (AHP) [26, 27]. The AHP was selected for weight assignment because of its effectiveness in structuring multilevel decision‐making problems. Unlike absolute scoring methods, AHP employs pairwise comparisons to determine relative importance, which offers greater precision in identifying nuanced clinical priorities. Furthermore, the methodology incorporates a mathematical consistency check for each comparison matrix to ensure that expert judgments are logically sound and to minimize potential bias. This structured approach provides a rigorous framework for translating qualitative expert consensus into a validated quantitative weight distribution, ensuring the reliability of the system for clinical and managerial prioritization.

### 2.2. Formation of the Research Team

To ensure robust project oversight and execution, two distinct committees were established. A multidisciplinary study management group responsible for day‐to‐day project management and research protocol execution was established, comprising 14 members: five senior professionals with ≥ 15 years of experience in neonatal care and quality indicator development, five associate senior professionals specializing in clinical implementation, one intermediate professional for project coordination, and three medical undergraduates for research support. Senior professionals provided overall research direction and framework design, associate senior professionals conducted indicator screening and validation, while coordination staff managed cross‐provincial data collection and preliminary analysis.

A study advisory group was also established to provide expert oversight and strategic guidance throughout the study. This group consisted of nine senior experts with distinguished backgrounds in neonatology, pediatric nursing, and medical quality management. The advisory group’s role was to provide guidance in three critical phases: (1) evaluate the initial indicator pool following literature synthesis, (2) review the Delphi questionnaire structure and content, and (3) validate the final indicator system and weight assignments to ensure both clinical relevance and scientific rigor.

#### 2.2.1. Phase 1: Indicator Development via Systematic Review

A comprehensive literature search was conducted across seven databases, including PubMed, Web of Science, Embase, Cochrane Library, CINAHL, and major Chinese databases (e.g., CNKI and Wanfang), from their inception to December 2023. This search strategy ensured that the initial indicator pool was grounded in both international evidence and domestic clinical guidelines relevant to the Chinese healthcare context.

Studies were included if they (1) focused on preterm infants (< 37 weeks gestation); (2) addressed discharge planning, transitional care processes, or postdischarge outcomes; and (3) described, evaluated, or proposed quality indicators, essential care components, or assessment tools relevant to the domain. To ensure a comprehensive evidence base, a wide range of study designs and publication types were included. These encompassed primary research (quantitative, qualitative, and mixed‐methods studies), systematic reviews, clinical practice guidelines, and expert consensus statements. We excluded conference abstracts, editorials, and studies without extractable indicator components.

Two researchers independently screened articles and extracted data from the included studies to develop a list of potential indicators. Disagreements were resolved through discussion with a third researcher. This list was subsequently discussed and refined by the study management group, incorporating relevant policy documents, to create the preliminary item pool for the Delphi survey.

#### 2.2.2. Phase 2: Consensus and Refinement via Delphi Survey

Purposive sampling was used to recruit 21 neonatal care experts from tertiary hospitals across four provinces (Sichuan, Beijing, Zhejiang, and Chongqing). This sample size aligns with methodological standards recommending 15–30 experts for hierarchical systems to ensure multidisciplinary coverage while maintaining consensus reliability (Cr) [22, 28]. To mitigate regional bias, provinces were strategically selected to represent clinical benchmarks across both eastern and western China. The panel included neonatologists, pediatric nurses, and quality management specialists, providing a balanced integration of clinical, educational, and managerial perspectives. To further minimize potential bias, experts were recruited from multiple independent institutions and across various career stages. All participants met strict inclusion criteria, requiring at least 5 years of relevant experience, a minimum of an undergraduate degree, and an intermediate or senior professional title in fields such as neonatology, nursing, or rehabilitation.

A two‐round Delphi survey was conducted between March and May 2024. The questionnaire required experts to rate the importance of each indicator on a five‐point Likert scale [28] (1 = “*least important*” to 5 = “*most important*”) and provided space for qualitative feedback to suggest modifications, additions, or deletions. After the first round, the research team analyzed the quantitative scores and qualitative comments. Indicators were retained if they achieved a mean importance score > 3.50, a coefficient of variation (CV) < 0.25, and a proportion of maximum scores > 20% [18]. Based on expert feedback, indicators were revised, merged, or newly generated. For the second round, experts received a summary of the group’s anonymous ratings from the first round and the revised indicator list, allowing them to reconsider their initial judgments. The process concluded after the second round when a high degree of consensus was achieved.

#### 2.2.3. Phase 3: Weight Assignment and Data Analysis

Data were analyzed using IBM SPSS Statistics 24.0 and Yaahp 11.0 software. Expert engagement was assessed by the questionnaire response rate. The authority and consensus of the expert panel were evaluated using the Cr coefficient, the CV to measure the concentration of opinions, and Kendall’s W coefficient to assess the consistency of expert rankings. A *p* value < 0.05 was considered statistically significant.

Following the finalization of the indicators through the Delphi process, the AHP was used to determine the relative weight of each indicator. Experts constructed pairwise comparison matrices to judge the relative importance of indicators within the same hierarchical level. The final weights were calculated, and a consistency test was performed for each matrix. A consistency ratio (CR) of less than 0.1 was considered acceptable, indicating a reliable judgment consistency [29].

### 2.3. Ethical considerations

This study was conducted in accordance with the principles of the Declaration of Helsinki, and the research protocol received ethical approval from the Ethics Committee of West China Second University Hospital (No. 2024–403). All participating experts provided informed consent prior to their involvement.

## 3. Results

### 3.1. Systematic Review and Initial Indicator Pool

The systematic literature search initially yielded 3902 articles. After removing duplicates and screening titles and abstracts, 896 articles were assessed for eligibility based on the inclusion and exclusion criteria. This process resulted in 68 studies meeting the inclusion criteria. These studies contributed 156 distinct indicator concepts across three primary domains: infant clinical readiness, healthcare system factors, and family/home environment factors. The evidence base demonstrated strong convergence around core transitional care components, including feeding competency, medication management, emergency preparedness, and family skill development. Following review and discussion by the research team, the initial item pool submitted for the first Delphi round consisted of 3 primary, 15 secondary, and 38 tertiary indicators.

### 3.2. Characteristics of the Expert Panel

A total of 21 experts participated in and completed both rounds of the Delphi survey, resulting in a 100% effective response rate. The panel was composed of experienced professionals, with 85.72% of experts having over 10 years of professional experience and 76.19% holding a master’s or doctoral degree. The majority held senior (38.10%) or associate senior (33.33%) professional titles (Table 1).

**TABLE 1:** Characteristics of the Delphi expert panel (*n* = 21).

Characteristics	Category	*n* (%)
Age (years)	≤ 35	3 (14.29)
	36–45	11 (52.38)
	46–55	6 (28.57)
	≥ 56	1 (4.76)

Professional experience (years)	≤ 10	3 (14.29)
	11–20	8 (38.10)
	21–30	10 (47.62)

Educational attainment	Bachelor degree	5 (23.81)
	Master degree	11 (52.38)
	Doctoral degree	5 (23.81)

Position	Director	2 (9.52)
	Deputy director	3 (14.29)
	Head nurse of the department	2 (9.52)
	Head nurse	5 (23.81)
	Deputy head nurse	2 (9.52)
	Other	7 (33.33)

Titles	Senior professional	8 (38.10)
	Associate senior professional	7 (33.33)
	Other	6 (28.57)

### 3.3. Expert Consensus and Indicator System Finalization

The expert agreement was high and consistent, as evidenced by Cr values of 0.967 and 0.964 for the first and second rounds, respectively (Table 2). A clear convergence of opinions was observed, with the CV for most indicators decreasing from the first round to the second. Furthermore, the consistency of expert rankings was statistically significant in both rounds, with Kendall’s W coefficients of 0.385 for Round 1 and 0.187 for Round 2 (both *p* < 0.05).

**TABLE 2:** Analysis of expert authority coefficients in the two Delphi rounds.

Experts	Round 1			Experts	Round 2		
	Cs	Ca	Cr		Cs	Ca	Cr
A1	1	0.9	0.95	B1	1	0.9	0.95
A2	1	1	1	B2	1	0.9	0.95
A3	1	1	1	B3	1	1	1
A4	1	1	1	B4	1	1	1
A5	1	1	1	B5	1	1	1
A6	1	0.9	0.95	B6	1	0.9	0.95
A7	0.8	0.9	0.85	B7	0.8	0.9	0.85
A8	1	1	1	B8	1	0.9	0.95
A9	1	1	1	B9	1	1	1
A10	1	1	1	B10	1	1	1
A11	1	0.9	0.95	B11	1	0.9	0.95
A12	1	1	1	B12	1	1	1
A13	1	1	1	B13	1	1	1
A14	1	0.8	0.9	B14	1	0.9	0.95
A15	0.8	1	0.9	B15	0.8	1	0.9
A16	1	0.9	0.95	B16	1	0.9	0.95
A17	0.8	0.9	0.85	B17	0.8	1	0.9
A18	1	1	1	B18	1	1	1
A19	1	1	1	B19	1	0.9	0.95
A20	1	1	1	B20	1	1	1
A21	1	1	1	B21	1	1	1

*Note:* Questionnaires in Round 1 were distributed to 21 experts (labeled as A), while those were sent in Round 2 to 21 experts (labeled as B).

This robust consensus process facilitated the progressive refinement of the indicator system. Following the first round, the framework was modified by revising 17 indicators and adding 12 new ones. After the second round, one secondary indicator (related management regulations) and three tertiary indicators (follow‐up system, transition care education system, and family‐centered care System) were removed. These deletions were based on a comprehensive analysis of quantitative results and qualitative feedback. Quantitatively, these indicators failed to meet the predefined consensus thresholds, demonstrating higher standard deviations and CVs compared to other items. Qualitatively, experts noted that these indicators represented macro policy frameworks rather than actionable clinical components. Moreover, the core elements of the deleted tertiary indicators were already integrated into more specific items within the system. Consequently, these indicators were excluded to enhance the system’s operational clarity and clinical feasibility, resulting in a finalized framework of 3 primary, 17 secondary, and 42 tertiary indicators.

### 3.4. Weight Distribution of the Finalized Indicators

**FIGURE 2:**
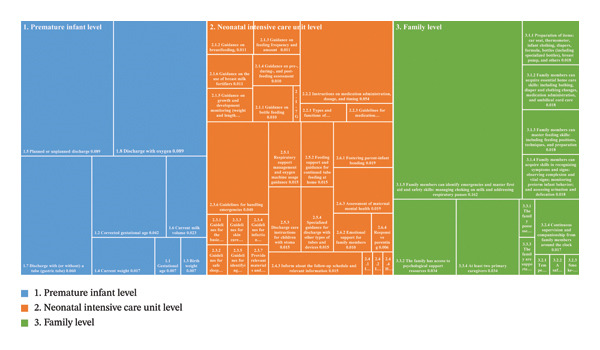
Hierarchical structure and weight distribution of the finalized indicator system.

Subsequently, the AHP was employed to determine the relative weight of each indicator within the finalized system. At the primary indicator level, the three domains—“Preterm Infant Level,” “Neonatal Intensive Care Unit Level,” and “Family Level”—were determined to be of equal importance, each receiving a weight of 0.333. Among the secondary indicators, the highest priority was given to “Transitional care readiness” (0.234) from the “Family Level” domain. This was followed by key indicators of the infant’s clinical complexity from the “Preterm Infant Level” domain, such as “Planned or unplanned transitional care” and “Transitional care with oxygen,” which both received a significant weight of 0.089. Finally, core hospital‐based preparatory components from the “NICU Level” domain, including “Feeding guidance,” “Instructions for medication use upon transitional care,” and “Guidance for home care,” were each weighted at 0.066. At the tertiary indicator level, the most critical item was identified as “Family emergency and first aid proficiency (choking and respiratory pauses),” which received the highest weight of 0.162. Other highly weighted tertiary indicators included “Instructions on medication administration, dosage, and timing” (0.054) and “Guidelines for handling emergencies” (0.040). A detailed breakdown of all indicators and their assigned weights is presented in Supporting Table 1, and a visual overview is provided in Figure 2.

## 4. Discussion

This study successfully developed and evaluated a comprehensive, multilevel management indicator system to enhance the quality of care for preterm infants during their transition from hospital to home. Through a rigorous methodology combining a systematic literature review with the Delphi technique and AHP, we established a scientifically robust framework comprising 3 primary, 17 secondary, and 42 tertiary indicators. This system addresses a critical gap in transitional care by providing a standardized tool for assessment, guidance, and quality improvement in this vulnerable patient population.

A principal finding is the balanced importance assigned to the three primary domains. The equal weighting (0.333 each) of the primary indicators (“Preterm Infant Level,” “Neonatal Intensive Care Unit Level,” and “Family Level”) provides robust, quantitative support for a holistic, family‐centered care model. This represents a significant advancement over traditional assessments that focus primarily on clinical stability, shifting the paradigm to an integrated approach that equally values institutional processes and family capabilities [9, 30]. This tripartite structure, endorsed by over 95% of the expert panel, operationalizes the consensus that a successful transition requires shared responsibility, demanding a seamless interface between the infant’s physiological maturity, the hospital’s preparatory actions, and the family’s capacity to provide complex care.

Beyond this overarching framework, the weighting of the secondary indicators delineates a clear and insightful hierarchy of clinical priorities. The paramount importance of family empowerment is highlighted by the finding that “Transitional care readiness” (0.234), an indicator under the “Family Level” domain, received the single highest weight, signifying that the expert panel views the family’s overall preparedness as the undisputed, most crucial determinant for a successful transition. This finding challenges the traditional, hospital‐centric view of discharge [31] and emphasizes that the ultimate goal of transitional care [32] is not simply to complete a checklist of medical tasks but to ensure that the family is confident and competent to manage care independently at home. Following this primary focus on the family, the system gives the next level of priority to the infant’s objective clinical condition. The significant weights assigned to indicators such as “planned or unplanned transitional care” and “Transitional care with oxygen” (both 0.089) underscore that the infant’s ongoing medical complexity is a critical factor. However, their weights, while substantial, are markedly lower than that of family readiness. This suggests that these clinical challenges are viewed not as standalone priorities but as the specific context that a well‐prepared family must be equipped to manage. Finally, at the foundational level of this hierarchy, are the instrumental, hospital‐based guidance components. The weights assigned to “Feeding guidance,” “Instructions for medication use upon transitional care,” and “Guidance for home care” (0.066 each) position them not as the end goals themselves but as the core educational pillars and essential tools [33] provided by the NICU to build the family readiness identified as the top priority.

The specific weighting of the tertiary‐level indicators further delineates a clear hierarchy of essential skills for caregivers. The exceptionally high weight assigned to “Family members can identify emergencies and master first aid and safety skills” (0.162) underscores its role as a decisive factor for infant safety during the transition from hospital to home. This finding reveals an identified gap in conventional discharge programs, which frequently prioritize routine caregiving knowledge such as feeding techniques over structured training in emergency recognition and intervention. Given that preterm infants are particularly susceptible to acute events such as apnea or choking [34, 35], families must be equipped to act as primary responders. The prioritization of this indicator suggests that experts perceive emergency response capability as a critical but often neglected component of current preparedness models, where educational content may be provided but competency is rarely verified. Consequently, there is an urgent need to reorient discharge preparation toward competency‐based and scenario‐oriented training to ensure that families can manage high‐stake clinical situations independently. This focus on proactive risk management is further reinforced by the substantial weights given to “Instructions on medication administration, dosage, and timing” (0.054) and “Guidelines for handling emergencies” (0.040). Together, these indicators reveal a consistent theme: Empowering families with concrete, life‐saving skills must be the foremost priority.

This pronounced emphasis on building tangible family capabilities, especially for high‐stake scenarios, is perhaps the system’s most significant contribution to clinical practice. Consequently, this indicator system provides healthcare teams with a standardized framework for systematic discharge assessment, which can reduce variation in clinical decision‐making and improve the quality of transitions. The weighted structure enables prioritized resource allocation, ensuring that the highest‐impact interventions, such as hands‐on emergency skills training, receive appropriate emphasis. For quality improvement initiatives, the system offers measurable benchmarks for evaluating the effectiveness of discharge planning. Healthcare organizations can utilize these indicators to identify opportunities for improvement, track progress over time, and compare performance across units or institutions.

The utility of this system is grounded in the study’s methodological rigor. Kendall’s W coefficients were statistically significant in both rounds (*p* < 0.05), indicating consistent agreement among experts. The reduction from 0.385 to 0.187 reflects greater dispersion in rankings as the indicator pool was narrowed and refined, while the stable Cr (Cr = 0.967 and 0.964) and the declining CV across most items indicate that expert opinions remained internally consistent throughout the process. However, several limitations warrant consideration. First, the expert panel was geographically concentrated in China, which may limit the direct international generalizability of the specific indicator weights. Nevertheless, the system’s core architecture, encompassing clinical stability, NICU discharge support, and family readiness, is rooted in universal transitional care principles. While the fundamental framework remains robust, its application in diverse healthcare environments may necessitate contextual adaptations to align with local healthcare structures, cultural norms, and resource availability. Furthermore, the indicator system requires prospective validation to evaluate its impact on clinical outcomes and the continuity of care. Future research will prioritize empirical testing in diverse clinical settings to assess the system’s effectiveness and feasibility in real‐world practice. Such studies will facilitate the refinement of indicators based on practical performance, ensuring that the system remains a robust and adaptive tool for neonatal transitional care management.

## 5. Conclusions

This study developed a comprehensive, evidence‐based indicator system for evaluating transitional care quality and transitional care readiness in preterm infants. The systematic methodology and high level of expert consensus support the framework’s content and face validity. By providing standardized assessment criteria with empirically derived weights, this system offers a structured framework that may help inform current discharge planning approaches and serve as a foundation for future quality improvement efforts. The indicator system’s emphasis on emergency preparedness, medication management, and family support capabilities provides a basis for targeted, individualized transitional care planning. However, its actual impact on family preparedness, readmission rates, and postdischarge outcomes remains to be established through prospective evaluation. Future work should focus on prospective implementation studies to assess the system’s clinical utility, validate its association with patient and family outcomes, adapt it for diverse healthcare contexts, and explore its integration into digital health platforms to facilitate clinical use.

## Author Contributions

Qing Meng: conceptualization, formal analysis, methodology, project administration, and writing–original draft. Fei Wang: methodology, software, and writing–original draft. Kefan Chen: data curation, software, and writing–original draft. Yingxin Li: validation and writing–reviewing and editing. Yuan Li: supervision, validation, and writing–reviewing and editing. Yanling Hu: conceptualization, funding acquisition, project administration, resources, writing–reviewing and editing, and supervision.

## Funding

This work was supported by the Sichuan Provincial Key Research and Development Program (Grant No. 2024YFFK0077).

## Conflicts of Interest

The authors declare no conflicts of interest.

## Supporting Information

Additional supporting information can be found online in the Supporting Information section.

## Supporting information


**Supporting Information** Supporting File 1. Search strategy for each database. Supporting Table 1. The finalized management indicator system with assigned indicator weights.

## Data Availability

The data that support the findings of this study are available on request from the corresponding author. The data are not publicly available due to privacy or ethical restrictions.

## References

[1] Ledinger D. , Nußbaumer-Streit B. , and Gartlehner G. , WHO Recommendations for Care of the Preterm or Low-Birth-Weight Infant, Gesundheitswesen. (2024) 86, no. 4, 289–293, 10.1055/a-2251-5686. 38467152 PMC11003242

[2] Ohuma E. O. , Moller A. B. , Bradley E. et al., National, Regional, and Global Estimates of Preterm Birth in 2020, With Trends From 2010: A Systematic Analysis, Lancet. (2023) 402, no. 10409, 1261–1271, 10.1016/s0140-6736(23)00878-4. 37805217

[3] Casavant S. G. , Cong X. , Moore J. , and Starkweather A. , Associations Between Preterm Infant Stress, Epigenetic Alteration, Telomere Length and Neurodevelopmental Outcomes: A Systematic Review, Early Human Development. (2019) 131, 63–74, 10.1016/j.earlhumdev.2019.03.003, 2-s2.0-85062653673. 30870624

[4] A Timely Arrival for Born Too Soon, Lancet. (2012) 380, 10.1016/s0140-6736(12)61970-9, 2-s2.0-84870467843. 23158234

[5] Rubens C. E. , Sadovsky Y. , Muglia L. , Gravett M. G. , Lackritz E. , and Gravett C. , Prevention of Preterm Birth: Harnessing Science to Address the Global Epidemic, Science Translational Medicine. (2014) 6, no. 262, 10.1126/scitranslmed.3009871, 2-s2.0-84910675031. 25391484

[6] Yardımcı-Lokmanoğlu B. N. , Livanelioğlu A. , Porsnok D. , Sırtbaş-Işık G. , Topal Y. , and Mutlu A. , Early Spontaneous Movements and Sensory Processing in Preterm Infants, American Journal of Occupational Therapy. (2023) 77, no. 3, 10.5014/ajot.2023.050096. 37352432

[7] Shaw R. J. , Givrad S. , Poe C. , Loi E. C. , Hoge M. K. , and Neurodevelopmental M. S. , Mental Health, and Parenting Issues in Preterm Infants, Children. (2023) 10, no. 9, 10.3390/children10091565. PMC10528009 37761526

[8] Connell A. , Knudsen K. , Marginean H. , and Raddish M. , Associations Between Feeding and Development in Preterm Infants in the NICU and Throughout the First Year of Life, Early Human Development. (2023) 177-178, 10.1016/j.earlhumdev.2023.105719. 36774728

[9] Chen H. and Dong L. , The Effect of Family Integrated Care on the Prognosis of Premature Infants, BMC Pediatrics. (2022) 22, no. 1, 10.1186/s12887-022-03733-0. PMC9675139 36403036

[10] Barkemeyer B. M. , Discharge Planning, Pediatric Clinics of North America. (2015) 62, no. 2, 545–556, 10.1016/j.pcl.2014.11.013, 2-s2.0-84931036288. 25836713

[11] Amsalu R. , Oltman S. P. , Baer R. J. , Medvedev M. M. , Rogers E. E. , and Jelliffe-Pawlowski L. , Incidence, Risk Factors, and Reasons for 30-Day Hospital Readmission Among Healthy Late Preterm Infants, Hospital Pediatrics. (2022) 12, no. 7, 639–649, 10.1542/hpeds.2021-006215. 35694876 PMC9997672

[12] Ong K. K. , Kennedy K. , Castañeda-Gutiérrez E. et al., Postnatal Growth in Preterm Infants and Later Health Outcomes: A Systematic Review, Acta Paediatrica. (2015) 104, no. 10, 974–986, 10.1111/apa.13128, 2-s2.0-84941906207. 26179961 PMC5054880

[13] Giuliani F. , Cheikh Ismail L. , Bertino E. et al., Monitoring Postnatal Growth of Preterm Infants: Present and Future, American Journal of Clinical Nutrition. (2016) 103, no. 2, 635s–647s, 10.3945/ajcn.114.106310, 2-s2.0-84956897536. 26791186 PMC6443302

[14] Schäfer N. , Karutz H. , and Schenk O. , The Need for Psychosocial Support of Parents of Children in Neonatal Care, Zeitschrift für Geburtshilfe und Neonatologie. (2017) 221, no. 05, 217–225, 10.1055/s-0043-110056, 2-s2.0-85020386611. 28591902

[15] Larsen J. N. , Hansson H. , Poorisrisak P. et al., Obstetric and Neonatal Conditions and Associations With Early Skin-to-Skin Contact—A Cross-Sectional Study, BMC Pregnancy and Childbirth. (2025) 25, no. 1, 10.1186/s12884-025-07811-w. PMC12224524 40604582

[16] Anjur K. I. and Darmstadt G. L. , Separation of Maternal and Newborn Care in US Hospitals: A Systemic Threat to Survival, Health and Well-Being, Health Systems & Reform. (2023) 9, no. 1, 10.1080/23288604.2023.2267255. 37890078

[17] Chen T. , Hu Y. , and Wan , Sichuan University West China Second Hospital. Analysis of the Reasons for Re-Admission of High-Risk Newborns in the Neonatal Department of a Grade III A Hospital and Corresponding Countermeasures, Chinese Journal of Practical Nursing. (2017) 33, 6–9, 10.3760/cma.j.issn.1672-7088.2017.z1.002.

[18] Gordijn S. J. , Beune I. M. , Thilaganathan B. et al., Consensus Definition of Fetal Growth Restriction: A Delphi Procedure, Ultrasound in Obstetrics and Gynecology. (2016) 48, no. 3, 333–339, 10.1002/uog.15884, 2-s2.0-84985032751. 26909664

[19] Griffith T. , Singh A. , Naber M. et al., Scoping Review of Interventions to Support Families With Preterm Infants Post-NICU Discharge, Journal of Pediatric Nursing. (2022) 67, e135–e149, 10.1016/j.pedn.2022.08.014. 36041959 PMC9729411

[20] Tubbs-Cooley H. L. , Pickler R. H. , Mara C. A. , Othman M. , Kovacs A. , and Mark B. A. , Hospital Magnet® Designation and Missed Nursing Care in Neonatal Intensive Care Units, Journal of Pediatric Nursing. (2017) 34, 5–9, 10.1016/j.pedn.2016.12.004, 2-s2.0-85008222808. 27955957

[21] Hu X. , Zhang Y. , Cao Y. , Huang G. , Hu Y. , and McArthur A. , Prevention of Neonatal Unplanned Extubations in the Neonatal Intensive Care Unit: A Best Practice Implementation Project, JBI Database of Systematic Reviews and Implementation Reports. (2017) 15, no. 11, 2789–2798, 10.11124/jbisrir-2016-003249, 2-s2.0-85050617597. 29135753

[22] Shang Z. , Use of Delphi in Health Sciences Research: A Narrative Review, Medicine (Baltimore). (2023) 102, no. 7, 10.1097/md.0000000000032829. PMC9936053 36800594

[23] Aagaard H. , Uhrenfeldt L. , Spliid M. , and Fegran L. , Parents’ Experiences of Transition When Their Infants are Discharged From the Neonatal Intensive Care Unit: A Systematic Review Protocol, JBI Database of Systematic Reviews and Implementation Reports. (2015) 13, no. 10, 123–132, 10.11124/jbisrir-2015-2287, 2-s2.0-85050579235. 26571288

[24] Osorio Galeano S. P. and Salazar Maya Á M. , Preparing Parents for Discharge From the Neonatal Unit, the Transition, and Care of Their Preterm Children at Home, Investigación y Educación en Enfermería. (2023) 41, 10.17533/udea.iee.v41n1e04. PMC10152913 37129352

[25] Tanaka M. C. , Bernardino F. B. S. , Braga P. P. , Alencastro L. , Gaíva M. A. M. , and Viera C. S. , Weaknesses in the Continuity of Care for Preterm Infants Discharged From the Neonatal Unit, Revista da Escola de Enfermagem da USP. (2024) 58, 10.1590/1980-220X-REEUSP-2023-0228en. PMC10946336 38497778

[26] Rybak Y. E. , Lai K. S. P. , Ramasubbu R. et al., Treatment-Resistant Major Depressive Disorder: Canadian Expert Consensus on Definition and Assessment, Depression and Anxiety. (2021) 38, no. 4, 456–467, 10.1002/da.23135. 33528865 PMC8049072

[27] Liu J. and Tang H. L. , Application of Fuzzy Analytic Hierarchy Process in Risk Assessment in Medicine Related Fields, Zhonghua Liuxingbingxue Zazhi. (2022) 43, no. 5, 766–770, 10.3760/cma.j.cn112338-20211130-00935. 35589586

[28] Tao X. , Zhang J. , Wang X. , Tao Y. , and Guo M. , Development of a Physical Literacy Assessment Framework for Chinese Preschool Children: A Delphi-AHP Approach, Frontiers in Public Health. (2025) 13, 10.3389/fpubh.2025.1650793. PMC12401909 40904921

[29] Oliveira C. , de Silva N. T. , Ungar W. J. et al., Health-Related Quality of Life in Neonates and Infants: A Conceptual Framework, Quality of Life Research. (2020) 29, no. 5, 1159–1168, 10.1007/s11136-020-02432-6. 31997081

[30] Cheong J. L. Y. , Burnett A. C. , Treyvaud K. , and Spittle A. J. , Early Environment and Long-Term Outcomes of Preterm Infants, Journal of Neural Transmission. (2020) 127, 1–8, 10.1007/s00702-019-02121-w. 31863172

[31] Bennett C. , Patients in a Globalized World: Keeping Hospitals in Perspective, Healthcare. (2003) 4, 50–56. 14660885

[32] Coleman E. A. and Min S. J. , Patients’ and Family Caregivers’ Goals for Care During Transitions Out of the Hospital, Home Health Care Services Quarterly. (2015) 34, no. 3-4, 173–184, 10.1080/01621424.2015.1095149, 2-s2.0-84949974147. 26496503

[33] Davidson J. E. , Aslakson R. A. , Long A. C. et al., Guidelines for Family-Centered Care in the Neonatal, Pediatric, and Adult ICU, Critical Care Medicine. (2017) 45, no. 1, 103–128, 10.1097/ccm.0000000000002169, 2-s2.0-85006409343. 27984278

[34] Belanger R. , Leroux D. , and Lefebvre P. , Supporting Caregivers of Children Born Prematurely in the Development of Language: A Scoping Review, Paediatrics and Child Health. (2021) 26, no. 1, e17–e24, 10.1093/pch/pxz124. 33542775 PMC7850271

[35] Dai K. , Fan X. , Shi H. et al., Application of Family-Centered Empowerment Model in Primary Caregivers of Premature Infants: A Quasi-Experimental Study, Frontiers in Pediatrics. (2023) 11, 10.3389/fped.2023.1137188. PMC10150083 37138569

