# Supplementary material for: Development and Evaluation of an Evidence‐Based Management Indicator System for Transitional Care in Preterm Infants Using a Delphi and AHP Approach
Source: J Nurs Manag. 2026 May 18;2026:2638144. doi: 10.1155/jonm/2638144 (PMC13182728; doi:10.1155/jonm/2638144)
Supplement: Supplementary file 1 — Supporting Information Supporting File 1. Search strategy for each database. Supporting Table 1. The finalized management indicator system with assigned indicator weights. [file JONM-2026-2638144-s001.docx]

Supporting File 1. Search strategy for each database.

Database(s): PubMed 2023/12

| # | Searches | Results |
| --- | --- | --- |
| 1 | ((((((Family nursing[MeSH Terms]) OR (Home Nursing[MeSH Terms])) OR (family caregivers[MeSH Terms])) OR (family*[Title/Abstract])) OR (home*[Title/Abstract])) OR (relative*[Title/Abstract])) OR (Informal caregiver*[Title/Abstract]) | 3,199,572 |
| 2 | (((((((((((Patient Discharge[MeSH Terms]) OR (discharge*[Title/Abstract])) OR (After Care[Title/Abstract])) OR (Patient Discharge[Title/Abstract])) OR (Discharge Planning[Title/Abstract])) OR (Early Patient Discharge[Title/Abstract])) OR (Transfer, Discharge[Title/Abstract])) OR (Patient Discharge Summaries[Title/Abstract])) OR (Admiss*[Title/Abstract])) OR (Admit*[Title/Abstract])) OR (Reamiss*[Title/Abstract])) OR (Readmit*[Title/Abstract]) | 781,889 |
| 3 | (((((((((Infant, Newborn[MeSH Terms]) OR (Infant, Newborn, Diseases[MeSH Terms])) OR (Infant Health[MeSH Terms])) OR (newborn*[Title/Abstract])) OR (infant*[Title/Abstract])) OR (premature infant[Title/Abstract])) OR (preterm[Title/Abstract])) OR (neonate*[Title/Abstract])) OR (NICU[Title/Abstract])) OR (neonatal intensive care unit[Title/Abstract]) | 1,145,774 |
| 4 | ((((((((Quality Indicators[MeSH Terms]) OR (Health Care[MeSH Terms])) OR (nursing sensitive quality indicator[Title/Abstract])) OR (quality indicator[Title/Abstract])) OR (patient safety indicator[Title/Abstract])) OR (outcomes indicator[Title/Abstract])) OR (nursing quality indexes[Title/Abstract])) OR (nursing care quality[Title/Abstract])) OR (standard of care[Title/Abstract]) | 1,362,700 |
| 5 | 1 AND 2 AND 3 AND 4:  (((((((((Family nursing[MeSH Terms]) OR (Home Nursing[MeSH Terms])) OR (family caregivers[MeSH Terms])) OR (family*[Title/Abstract])) OR (home*[Title/Abstract])) OR (relative*[Title/Abstract])) OR (Informal caregiver*[Title/Abstract])) AND ((((((((((((Patient Discharge[MeSH Terms]) OR (discharge*[Title/Abstract])) OR (After Care[Title/Abstract])) OR (Patient Discharge[Title/Abstract])) OR (Discharge Planning[Title/Abstract])) OR (Early Patient Discharge[Title/Abstract])) OR (Transfer, Discharge[Title/Abstract])) OR (Patient Discharge Summaries[Title/Abstract])) OR (Admiss*[Title/Abstract])) OR (Admit*[Title/Abstract])) OR (Reamiss*[Title/Abstract])) OR (Readmit*[Title/Abstract]))) AND ((((((((((Infant, Newborn[MeSH Terms]) OR (Infant, Newborn, Diseases[MeSH Terms])) OR (Infant Health[MeSH Terms])) OR (newborn*[Title/Abstract])) OR (infant*[Title/Abstract])) OR (premature infant[Title/Abstract])) OR (preterm[Title/Abstract])) OR (neonate*[Title/Abstract])) OR (NICU[Title/Abstract])) OR (neonatal intensive care unit[Title/Abstract]))) AND (((((((((Quality Indicators[MeSH Terms]) OR (Health Care[MeSH Terms])) OR (nursing sensitive quality indicator[Title/Abstract])) OR (quality indicator[Title/Abstract])) OR (patient safety indicator[Title/Abstract])) OR (outcomes indicator[Title/Abstract])) OR (nursing quality indexes[Title/Abstract])) OR (nursing care quality[Title/Abstract])) OR (standard of care[Title/Abstract])) | 1,248 |

Database(s):Web of Science 2023/12

| # | Searches | Results |
| --- | --- | --- |
| 1 | “Family nursing” OR “Home Nursing” OR “family caregivers” OR family* OR home* OR relative* OR “Informal caregiver*” (Title) | 697,301 |
| 2 | “Family nursing” OR “Home Nursing” OR “family caregivers” OR family* OR home* OR relative* OR “Informal caregiver*” (Abstract) | 4,923,994 |
| 3 | #1 OR #2 | 5,274,589 |
| 4 | “Patient Discharge” OR discharge* OR “After Care” OR “Patient Discharge” OR “Discharge Planning” OR “Early Patient Discharge” OR “Transfer, Discharge” OR “Patient Discharge Summaries” OR Admiss* OR Admit* OR Reamiss* OR Readmit* (Title) | 199,420 |
| 5 | “Patient Discharge” OR discharge* OR “After Care” OR “Patient Discharge” OR “Discharge Planning” OR “Early Patient Discharge” OR “Transfer, Discharge” OR “Patient Discharge Summaries” OR Admiss* OR Admit* OR Reamiss* OR Readmit* (Abstract) | 1,030,982 |
| 6 | #4 OR #5 | 1,114,431 |
| 7 | “Infant, Newborn” OR “Infant, Newborn, Diseases” OR “Infant Health” OR “newborn*” OR “infant*” OR “premature infant” OR “preterm” or “neonate*” OR “NICU” OR “neonatal intensive care unit” (Title) | 354,468 |
| 8 | “Infant, Newborn” OR “Infant, Newborn, Diseases” OR “Infant Health” OR “newborn*” OR “infant*” OR “premature infant” OR “preterm” or “neonate*” OR “NICU” OR “neonatal intensive care unit” (Abstract) | [547,532](https://webofscience.clarivate.cn/wos/woscc/summary/2c339ea0-7954-4b8a-8f08-aad0ca637c92-0174afe4d9/relevance/1) |
| 9 | #7 OR #8 | [727,255](https://webofscience.clarivate.cn/wos/woscc/summary/d4ab00b3-c010-4c27-8b4e-edfe153c274d-0174afe86f/relevance/1) |
| 10 | “Quality Indicators” OR “Health Care” OR “nursing sensitive quality indicator” OR “quality indicator” OR “patient safety indicator” OR “outcomes indicator” OR “nursing quality indexes” OR “nursing care quality” OR “standard of care” (Title) | 115,198 |
| 11 | “Quality Indicators” OR “Health Care” OR “nursing sensitive quality indicator” OR “quality indicator” OR “patient safety indicator” OR “outcomes indicator” OR “nursing quality indexes” OR “nursing care quality” OR “standard of care” (Abstract) | 360,862 |
| 12 | #10 OR #11 | 427,554 |
| 13 | #3 AND #6 AND #9 AND #12 | 404 |

Database(s): Embase 2023/12

| # | Searches | Results |
| --- | --- | --- |
| 1 | family nursing/ | 1,652 |
| 2 | home care/ | 79,371 |
| 3 | caregiver/ | 165,488 |
| 4 | (family* or home* or relative* or Informal caregiver*).ab,ti. | 4,546,474 |
| 5 | 1 or 2 or 3 or 4 | 4,656,559 |
| 6 | hospital discharge/ | 225,962 |
| 7 | (discharge* or After Care or Patient Discharge or Discharge Planning or Early Patient Discharge or Transfer, Discharge or Patient Discharge Summaries or Admiss* or Admit* or Reamiss* or Readmit*).ab,ti. | 1,534,194 |
| 8 | 6 or 7 | 1,602,672 |
| 9 | newborn/ | 677,607 |
| 10 | newborn disease/ | 26,289 |
| 11 | child health/ | 41,839 |
| 12 | (newborn* or infant* or premature infant or preterm or neonate* or NICU or neonatal intensive care unit).ab,ti. | 946,402 |
| 13 | 9 or 10 or 11 or 12 | 1,308,241 |
| 14 | health care/ | 152,679 |
| 15 | (Quality Indicators or nursing sensitive quality indicator or quality indicator or patient safety indicator or outcomes indicator or nursing quality indexes or nursing care quality or standard of care).ab,ti. | 204,528 |
| 16 | 14 or 15 | 355,400 |
| 17 | 5 and 8 and 13 and 16 | 710 |

Database(s): Cochrane Library 2023/12

| # | Searches | Results |
| --- | --- | --- |
| 1 | ("Family nursing" or "Home Nursing" or "family caregivers" or family* or home* or relative* or "Informal caregiver*").ti,ab. | 2,211 |
| 2 | ("Patient Discharge" or discharge* or "After Care" or "Patient Discharge" or "Discharge Planning" or "Early Patient Discharge" or "Transfer, Discharge" or "Patient Discharge Summaries" or Admiss* or Admit* or Reamiss* or Readmit*).ti,ab. | 941 |
| 3 | ("Infant, Newborn" or "Infant, Newborn, Diseases" or "Infant Health" or "newborn*" or "infant*" or "premature infant" or "preterm" or "neonate*" or "NICU" or "neonatal intensive care unit").ti,ab. | 1,215 |
| 4 | ("Quality Indicators" or "Health Care" or "nursing sensitive quality indicator" or "quality indicator" or "patient safety indicator" or "outcomes indicator" or "nursing quality indexes" or "nursing care quality" or "standard of care").ti,ab. | 797 |
| 5 | 1 and 2 and 3 and 4 | 7 |

Database(s): CINAHL 2023/12

| # | Searches | Results |
| --- | --- | --- |
| 1 | [MH "Family Nursing"](https://research.ebsco.com/search/results?db=cul&expanders=concept&limiters=None&q=MH%20) | 2,134 |
| 2 | [MH "Home Nursing"](https://research.ebsco.com/search/results?db=cul&expanders=concept&limiters=None&q=MH%20) | 3,876 |
| 3 | [MH "Family Caregiver Status (Iowa NOC)"](https://research.ebsco.com/search/results?db=cul&expanders=concept&limiters=None&q=MH%20) | 664 |
| 4 | [XB family* OR home* OR relative* OR “Informal caregiver*”](https://research.ebsco.com/search/results?db=cul&expanders=concept&limiters=None&q=XB%20family*%20OR%20home*%20OR%20relative*%20OR%20%E2%80%9CInformal%20caregiver*%E2%80%9D&qm=W3sidmFsdWUiOiJmYW1pbHkqIE9SIGhvbWUqIE9SIHJlbGF0aXZlKiBPUiDigJxJbmZvcm1hbCBjYXJlZ2l2ZXIq4oCdIiwidHlwZSI6ImZpZWxkIiwiY29kZSI6IlhCIn1d&searchMode=boolean&sort=relevance&userDirectAction=true&isDashboardExpanded=true) | 641,479 |
| 5 | [S1 OR S2 OR S3 OR S4](https://research.ebsco.com/search/results?combinedSearchQueryId=sq:c935eb32-36a6-4d3b-842e-0f38dc09ef76&db=cul&expanders=concept&limiters=None&q=S1%20OR%20S2%20OR%20S3%20OR%20S4&searchMode=boolean&sort=relevance&userDirectAction=true&isDashboardExpanded=true) | 644,166 |
| 6 | [MH "Patient Discharge"](https://research.ebsco.com/search/results?db=cul&expanders=concept&limiters=None&q=MH%20) | 23,619 |
| 7 | XB discharge* OR “After Care” OR “Patient Discharge” OR “Discharge Planning” OR “Early Patient Discharge” OR “Transfer, Discharge” OR “Patient Discharge Summaries” OR Admiss* OR Admit* OR Reamiss* OR Readmit* | 218,228 |
| 8 | [S6 OR S7](https://research.ebsco.com/search/results?combinedSearchQueryId=sq:f9157ac9-d1e5-4821-a816-d66b89dd629a&db=cul&expanders=concept&limiters=None&q=S6%20OR%20S7&searchMode=boolean&sort=relevance&userDirectAction=true&isDashboardExpanded=true) | 224,581 |
| 9 | [MH "Infant, Newborn"](https://research.ebsco.com/search/results?db=cul&expanders=concept&limiters=None&q=MH%20) | 145,452 |
| 10 | [MH "Infant, Newborn, Diseases"](https://research.ebsco.com/search/results?db=cul&expanders=concept&limiters=None&q=MH%20) | 4,526 |
| 11 | [XB “Infant Health” OR “newborn*” OR “infant*” OR “premature infant” OR “preterm” or “neonate*” OR “NICU” OR “neonatal intensive care unit”](https://research.ebsco.com/search/results?db=cul&expanders=concept&limiters=None&q=XB%20%E2%80%9CInfant%20Health%E2%80%9D%20OR%20%E2%80%9Cnewborn*%E2%80%9D%20OR%20%E2%80%9Cinfant*%E2%80%9D%20OR%20%E2%80%9Cpremature%20infant%E2%80%9D%20OR%20%E2%80%9Cpreterm%E2%80%9D%20or%20%E2%80%9Cneonate*%E2%80%9D%20OR%20%E2%80%9CNICU%E2%80%9D%20OR%20%E2%80%9Cneonatal%20intensive%20care%20unit%E2%80%9D&qm=W3sidmFsdWUiOiLigJxJbmZhbnQgSGVhbHRo4oCdIE9SIOKAnG5ld2Jvcm4q4oCdIE9SIOKAnGluZmFudCrigJ0gT1Ig4oCccHJlbWF0dXJlIGluZmFudOKAnSBPUiDigJxwcmV0ZXJt4oCdIG9yIOKAnG5lb25hdGUq4oCdIE9SIOKAnE5JQ1XigJ0gT1Ig4oCcbmVvbmF0YWwgaW50ZW5zaXZlIGNhcmUgdW5pdOKAnSIsInR5cGUiOiJmaWVsZCIsImNvZGUiOiJYQiJ9XQ==&searchMode=boolean&sort=relevance&userDirectAction=true&isDashboardExpanded=true) | 183,576 |
| 12 | [S9 OR S10 OR S11](https://research.ebsco.com/search/results?combinedSearchQueryId=sq:5125f272-cf8f-4ca8-abc8-1585743ba54f&db=cul&expanders=concept&limiters=None&q=S9%20OR%20S10%20OR%20S11&searchMode=boolean&sort=relevance&userDirectAction=true&isDashboardExpanded=true) | 255,169 |
| 13 | XB “Quality Indicators” OR “Health Care” OR “nursing sensitive quality indicator” OR “quality indicator” OR “patient safety indicator” OR “outcomes indicator” OR “nursing quality indexes” OR “nursing care quality” OR “standard of care” | 252,792 |
| 14 | [S5 AND S8 AND S12 AND S13](https://research.ebsco.com/search/results?combinedSearchQueryId=sq:46022bd8-f088-495d-89ca-b7639c13468d&db=cul&expanders=concept&limiters=None&q=S5%20AND%20S8%20AND%20S12%20AND%20S13&searchMode=boolean&sort=relevance&userDirectAction=true&isDashboardExpanded=true) | 423 |

Supporting Table 1. The finalized management indicator system with assigned indicator weights.

| Indicator classification | Indicator details | Importance Score  $\text{(Mean ± SD)}$ | $\mathbf{CV}$ | $\mathbf{W}$ | $\mathbf{F}$ | $\mathbf{P}$ |
| --- | --- | --- | --- | --- | --- | --- |
| Primary indicator | 1. Premature infant level | 4.95±0.22 | 0.04 | 0.333 | 0.952 | <0.001** |
|  | 2. Neonatal intensive care unit level | 4.95±0.22 | 0.04 | 0.333 | 1.000 |  |
|  | 3. Family level | 5.00±0.00 | 0.00 | 0.333 | 0.952 |  |
| Secondary indicator | 1.1 Gestational age | 4.86±0.48 | 0.10 | 0.007 | 0.904 | 0.039* |
|  | 1.2 Corrected gestational age | 4.81±0.40 | 0.08 | 0.042 | 0.809 |  |
|  | 1.3 Birth weight | 4.90±0.30 | 0.06 | 0.007 | 0.904 |  |
|  | 1.4 Current weight | 4.95±0.22 | 0.04 | 0.017 | 0.952 |  |
|  | 1.5 Planned or unplanned transitional care | 4.86±0.36 | 0.07 | 0.089 | 0.857 |  |
|  | 1.6 Current milk volume | 4.86±0.36 | 0.07 | 0.023 | 0.857 |  |
|  | 1.7 Transitional care with (or without) a tube (gastric tube) | 4.90±0.30 | 0.06 | 0.060 | 0.904 |  |
|  | 1.8 Transitional care with oxygen | 4.90±0.30 | 0.06 | 0.089 | 0.904 |  |
|  | 2.1 Feeding guidance | 5.00±0.00 | 0.00 | 0.066 | 1.000 | 0.063 |
|  | 2.2 Instructions for medication use upon transitional care | 4.90±0.30 | 0.06 | 0.066 | 0.904 |  |
|  | 2.3 Guidance for home care | 5.00±0.00 | 0.00 | 0.066 | 1.000 |  |
|  | 2.4 Follow-up guidance | 4.90±0.30 | 0.06 | 0.021 | 0.904 |  |
|  | 2.5 Guidance for special medical care needs | 4.95±0.22 | 0.04 | 0.059 | 0.952 |  |
|  | 2.6 Psychological support | 4.81±0.40 | 0.08 | 0.054 | 0.809 |  |
|  | 3.1 Transitional care readiness | 4.95±0.22 | 0.04 | 0.234 | 0.952 | 0.052 |
|  | 3.2 Home environment | 4.86±0.36 | 0.07 | 0.024 | 0.857 |  |
|  | 3.3 Family support | 5.00±0.00 | 0.00 | 0.076 | 1.000 |  |
| Tertiary indicator | 2.1.1 Guidance on bottle feeding | 5.00±0.00 | 0.00 | 0.010 | 1.000 | 0.006** |
|  | 2.1.2 Guidance on breastfeeding | 4.95±0.22 | 0.04 | 0.011 | 0.952 |  |
|  | 2.1.3 Guidance on feeding frequency and amount | 4.95±0.22 | 0.04 | 0.011 | 0.952 |  |
|  | 2.1.4 Guidance on pre-, during-, and post-feeding assessment | 4.95±0.22 | 0.04 | 0.010 | 0.952 |  |
|  | 2.1.5 Guidance on growth and development monitoring (weight and length measurement) | 4.95±0.22 | 0.04 | 0.011 | 0.952 |  |
|  | 2.1.6 Guidance on the use of breast milk fortifiers | 4.90±0.30 | 0.06 | 0.011 | 0.904 |  |
|  | 2.1.7 Guidance on the cleaning and disinfection of feeding equipment | 4.81±0.51 | 0.11 | 0.002 | 0.857 |  |
|  | 2.2.1 Types and functions of medications to continue after transitional care | 4.95±0.22 | 0.04 | 0.006 | 0.952 | <0.001** |
|  | 2.2.2 Instructions on medication administration, dosage, and timing | 5.00±0.00 | 0.00 | 0.054 | 1.000 |  |
|  | 2.2.3 Guidelines for medication storage | 4.81±0.40 | 0.08 | 0.006 | 0.809 |  |
|  | 2.3.1 Guidelines for the basic principles of daily care | 4.71±0.64 | 0.14 | 0.004 | 0.809 | <0.001** |
|  | 2.3.2 Guidelines for safe sleep | 4.86±0.36 | 0.07 | 0.004 | 0.857 |  |
|  | 2.3.3 Guidelines for skin care | 4.76±0.44 | 0.09 | 0.004 | 0.761 |  |
|  | 2.3.4 Guidelines for infection prevention | 4.86±0.36 | 0.07 | 0.004 | 0.857 |  |
|  | 2.3.5 Guidelines for preterm infants: identifying common behaviors, issues and interventions | 4.90±0.30 | 0.06 | 0.004 | 0.904 |  |
|  | 2.3.6 Guidelines for handling emergencies | 5.00±0.00 | 0.00 | 0.040 | 1.000 |  |
|  | 2.3.7 Provide relevant materials and resources | 4.76±0.44 | 0.09 | 0.004 | 0.761 |  |
|  | 2.4.1 Inform about vaccination information | 4.76±0.44 | 0.09 | 0.002 | 0.761 | <0.001** |
|  | 2.4.2 Inform about child health protection card information | 4.81±0.40 | 0.08 | 0.002 | 0.809 |  |
|  | 2.4.3 Inform about the follow-up schedule and relevant information | 4.95±0.22 | 0.04 | 0.015 | 0.952 |  |
|  | 2.4.4 High-risk premature infant follow-up platform | 4.62±0.50 | 0.11 | 0.002 | 0.619 |  |
|  | 2.5.1 Respiratory support management and oxygen machine usage guidance | 4.95±0.22 | 0.04 | 0.015 | 0.952 | <0.001** |
|  | 2.5.2 Feeding support and guidance for continued tube feeding at home | 5.00±0.00 | 0.00 | 0.015 | 1.000 |  |
|  | 2.5.3 Transitional care instructions for preterm infants with stoma | 5.00±0.00 | 0.00 | 0.015 | 1.000 |  |
|  | 2.5.4 Specialized guidance for transitional care with other types of tubes and devices | 4.95±0.22 | 0.04 | 0.015 | 0.952 |  |
|  | 2.6.1 Fostering parent-infant bonding | 4.90±0.30 | 0.06 | 0.019 | 0.904 | 0.004** |
|  | 2.6.2 Emotional support from family members | 4.90±0.30 | 0.06 | 0.010 | 0.904 |  |
|  | 2.6.3 Assessment of maternal mental health | 4.76±0.44 | 0.09 | 0.019 | 0.761 |  |
|  | 2.6.4 Responsive parenting | 4.67±0.48 | 0.10 | 0.006 | 0.667 |  |
|  | 3.1.1 Preparation of items: car seat, thermometer, infant clothing, diapers, formula, bottles (e.g., specialized bottles), breast pump, etc. | 4.95±0.22 | 0.04 | 0.018 | 0.952 | <0.001** |
|  | 3.1.2 Family mastery of essential home care skills: bathing, diaper and clothing changes, medication, and umbilical cord care | 5.00±0.00 | 0.00 | 0.018 | 1.000 |  |
|  | 3.1.3 Family mastery of feeding skills: positions, techniques, and preparation | 5.00±0.00 | 0.00 | 0.018 | 1.000 |  |
|  | 3.1.4 Family mastery of skills in recognizing symptoms and signs: observing complexion, vital signs, behavior, urination and defecation | 5.00±0.00 | 0.00 | 0.018 | 1.000 |  |
|  | 3.1.5 Family emergency and first aid proficiency (choking and respiratory pauses) | 5.00±0.00 | 0.00 | 0.162 | 1.000 |  |
|  | 3.2.1 Proper adjustment of temperature, humidity and lighting | 4.90±0.30 | 0.06 | 0.002 | 0.904 | <0.001**  <0.001** |
|  | 3.2.2 Safe, tranquil sleeping environment, (e.g., separate crib) | 4.86±0.36 | 0.07 | 0.002 | 0.857 |  |
|  | 3.2.3 Smoke-free and odorless | 4.86±0.36 | 0.07 | 0.002 | 0.857 |  |
|  | 3.2.4 24-Hour family supervision and care | 5.00±0.00 | 0.00 | 0.017 | 1.000 |  |
|  | 3.3.1 The family possesses financial support resources | 4.86±0.36 | 0.07 | 0.004 | 0.857 |  |
|  | 3.3.2 The family possesses psychological support resources | 4.90±0.30 | 0.06 | 0.034 | 0.904 |  |
|  | 3.3.3 The family possesses social support resources | 4.81±0.40 | 0.08 | 0.004 | 0.809 |  |
|  | 3.3.4 At least two primary caregivers | 4.67±0.48 | 0.10 | 0.034 | 0.667 |  |

SD: Standard Deviation; CV: Coefficient of Variation; W: Kendall’s W coefficient; F: Frequency of Full Marks; **P* <0.05;***P* <0.01

Supporting Table 2: Standardized instructions on Individual Indicators within the Index System

| Indicator classification | Indicator details | Standardized instructions |
| --- | --- | --- |
| Primary indicator | 1. Premature infant level | Definition: Preterm infants’ maturity, stability, and discharge eligibility. |
|  | 2. Neonatal intensive care unit level | Definition: NICU’s capacity to support safe discharge transitions. |
|  | 3. Family level | Definition: Family’s readiness to care for preterm infants after discharge. |
| Secondary indicator | 1.1 Gestational age | Definition: Gestational age at birth (weeks + days).  Assessment standard: ≥28 weeks, and <37 weeks. [1] |
|  | 1.2 Corrected gestational age | Definition: Gestational age adjusted for preterm birth.  (postnatal age + gestational age at birth) .  Assessment standard: ≥34 weeks (standard discharge threshold). [1] |
|  | 1.3 Birth weight | Definition: Body weight of the preterm infant at birth (g). |
|  | 1.4 Current weight | Definition: Body weight of the preterm infant at discharge (g) and weight gain rate.  Assessment standard:   1. Measured daily for 3 consecutive days before discharge. 2. ≥1800-2000g with stable weight gain (≥15g/kg/d). [1] |
|  | 1.5 Planned or unplanned discharge | Definition: Classification of discharge type.  Assessment standard:   1. planned = meets clinical stability criteria; 2. unplanned = due to family request/medical emergency. [1] |
|  | 1.6 Current milk volume | Definition: Daily total enteral feeding volume at discharge (ml/kg/d).  Assessment standard:   1. Recorded in feeding logs for 48 consecutive hours. 2. 150-180ml/kg/d with no vomiting/aspiration. [2] |
|  | 1.7 Discharge with (or without) a tube (gastric tube) | Definition: Whether the preterm infant requires a tube for feeding at discharge. |
|  | 1.8 Discharge with oxygen | Definition: Whether the preterm infant requires oxygen support at discharge.  Assessment standard: Without oxygen inhalation or using home oxygen therapy equipment, SpO2 can be maintained at 0.90-0.95. [1] |
|  | 2.1 Feeding guidance | Definition: NICU’s education for families on preterm infant feeding.  Assessment standard:   1. Covers core skills (breastfeeding position, formula preparation, feeding frequency, choking first aid, etc.); 2. Taught via "explain-demonstrate-family practice"; 3. Family completes 2 independent correct feedings. |
|  | 2.2 Instructions for medication use upon discharge | Definition: NICU’s guidance on post-discharge medication administration.  Assessment standard:   1. Provides written plan (name, dose, route, frequency); 2. Family demonstrates correct administration; 3. Family identifies 2 common side effect. |
|  | 2.3 Guidance for home care | Definition: NICU’s education on daily home care for preterm infants.  Assessment standard:   1. Covers tasks (skin care, temperature monitoring, diaper care, etc.); 2. Family describes key steps. |
|  | 2.4 Follow-up guidance | Definition: NICU’s guidance on post-discharge follow-up.  Assessment standard: 1st follow-up within 72h, weekly for 4w. |
|  | 2.5 Guidance for special medical care needs | Definition: NICU’s guidance on managing preterm infants’ special care needs.  Assessment standard: Customizes care plan (e.g., gastrostomy, apnea monitor). |
|  | 2.6 Psychological support | Definition: NICU’s psychological support for family caregivers. |
|  | 3.1 Discharge readiness | Definition: The family’s overall readiness to care for the discharge preterm infant.  Assessment standard: Evaluated via Early Infant Parent Training and Family Preparation Checklist. [1] |
|  | 3.2 Home environment | Definition: Suitability of the home environment for preterm infant care. [1] |
|  | 3.3 Family support | Definition: Family’s access to support resources for preterm infant care. [1] |
| Tertiary indicator | 2.1.1 Guidance on bottle feeding | Definition: NICU’s education on bottle-feeding techniques for preterm infants.  Assessment standard:   1. Covers nipple flow selection (preterm-specific slow flow), feeding position (semi-upright), and pace control (pause every 5-10ml); 2. Family demonstrates 1 complete, spill-free bottle-feeding. [2] |
|  | 2.1.2 Guidance on breastfeeding | Definition: NICU’s guidance on breastfeeding adaptation for preterm infants. Assessment standard:   1. Covers kangaroo care breastfeeding, latch assistance, and expressed breast milk supplementation; 2. Family achieves effective latch with guidance. [4] |
|  | 2.1.3 Guidance on feeding frequency and amount | Definition: NICU’s instruction on preterm infant feeding schedule and volume.  Assessment standard:  Recommends frequency (every 2-3h) and volume (gradually increases to 150-180ml/kg/d);  Family correctly records feeding volume for 2 consecutive days. [3] |
|  | 2.1.4 Guidance on pre-, during-, and post-feeding assessment | Definition: NICU’s education on feeding-related assessment for preterm infants.  Assessment standard:  Pre-feeding: Check gastric residual (≤2ml/kg);  During-feeding: Monitor respiratory rate/skin color;  Post-feeding: Burp and keep upright for 15min. [4] |
|  | 2.1.5 Guidance on growth and development monitoring (weight and length measurement) | Definition: NICU’s guidance on preterm infant growth tracking.  Assessment standard:   1. Teaches weight (daily) and length (weekly) measurement methods; 2. Family correctly uses measurement tools. |
|  | 2.1.6 Guidance on the use of breast milk fortifiers | Definition: NICU’s instruction on breast milk fortification for preterm infants.  Assessment standard: Family can mix and use the supplements correctly. |
|  | 2.1.7 Guidance on the cleaning and disinfection of feeding equipment | Definition: NICU’s guidance on hygiene of preterm infant feeding tools.  Assessment standard: Teaches cleaning and disinfection steps. |
|  | 2.2.1 Types and functions of medications to continue after discharge | Definition: NICU’s explanation of post-discharge medications for preterm infants.  Assessment standard:   1. Clarifies drug types (e.g., vitamin supplements) and therapeutic purposes; 2. Family correctly identifies each medication’s function. |
|  | 2.2.2 Instructions on medication administration, dosage, and timing | Definition: NICU’s guidance on post-discharge medication use for preterm infants.  Assessment standard: Specifies administration route (oral), dosage (weight-based), and timing. |
|  | 2.2.3 Guidelines for medication storage | Definition: NICU’s instruction on proper storage of post-discharge medications.  Assessment standard:   1. Clarifies storage conditions (e.g., refrigeration for 2-8℃ drugs); 2. Family identifies correct storage location. |
|  | 2.3.1 Guidelines for the basic principles of daily care | Definition: NICU’s guidance on core daily care principles for preterm infants.  Assessment standard:   1. Covers 4 key areas (temperature maintenance, diaper care, umbilical cord care, and oral hygiene); 2. family demonstrates correct application of at least 2 principles. [4] |
|  | 2.3.2 Guidelines for safe sleep | Definition: NICU’s education on safe sleep practices for preterm infants. |
|  | 2.3.3 Guidelines for skin care | Definition: NICU guidance on preterm infant skin care.  Assessment standard: Covers gentle cleansing, moisturization, and pressure care. |
|  | 2.3.4 Guidelines for infection prevention | Definition: NICU’s guidance on reducing infection risk in preterm infants.  Assessment standard: Teaches hand hygiene, visitor screening, and disinfection of care items. |
|  | 2.3.5 Guidelines for identifying common behaviors, issues, and interventions for premature infants | Definition: NICU’s education on recognizing preterm infant behaviors and managing common issues.  Assessment standard: Covers 3 common behaviors (e.g., rooting, fussing) and 2 common issues (e.g., regurgitation, jaundice). [1] |
|  | 2.3.6 Guidelines for handling emergencies | Definition: NICU’s guidance on recognizing and managing preterm infant emergencies.  Assessment standard: Covers 3 key emergencies (apnea, seizure, respiratory distress) and their initial interventions. |
|  | 2.3.7 Provide relevant materials and resources | Definition: NICU’s provision of educational materials and support resources for preterm infant care.  Assessment standard: Provides discharge handbook, emergency contacts, and online support; family confirms receipt and understanding. |
|  | 2.4.1 Inform about vaccination information | Definition: NICU guidance on the application, acquisition, and proper use of the child health protection card.  Assessment standard: Clarifies corrected age-based vaccine timing, contraindications, and catch-up schedule. [1] |
|  | 2.4.2 Inform about child health protection card information | Definition: NICU guidance on using child health protection card.  Assessment standard: Explains card registration, health record documentation, and clinic visit procedures; family demonstrates ability to locate card information. |
|  | 2.4.3 Inform about the follow-up schedule and relevant information | Definition: NICU’s instruction on preterm infant post-discharge follow-up plan.  Assessment standard: Specifies follow-up timing, required assessments (growth, neurodevelopment), and referral process. [1] |
|  | 2.4.4 High-risk premature infant follow-up platform | Definition: NICU’s introduction to a dedicated follow-up platform for high-risk preterm infants.  Assessment standard: Explains core platform functions (remote monitoring, consultation, records), family registers and demonstrates basic navigation. |
|  | 2.5.1 Respiratory support management and oxygen machine usage guidance | Definition: NICU’s instruction on preterm infant respiratory support and safe operation of home oxygen therapy equipment.  Assessment standard: Explains home oxygen machine setup, SpO₂ monitoring, and alarm troubleshooting |
|  | 2.5.2 Feeding support and guidance for continued tube feeding at home | Definition: NICU’s education on enteral tube feeding techniques and care for preterm infants at home.  Assessment standard: Covers tube maintenance, feeding preparation, and recognition of complications, family demonstrates proper feeding procedure. |
|  | 2.5.3 Discharge care instructions for children with stoma | Definition: NICU’s guidance on home care and management of infant stoma.  Assessment standard: Explains stoma cleaning, skin care, and bag changes. |
|  | 2.5.4 Specialized guidance for discharge with other types of tubes and devices | Definition: NICU’s education on home care for preterm infants with indwelling tubes or medical devices.  Assessment standard: Covers device maintenance, infection prevention, and emergency removal. |
|  | 2.6.1 Fostering parent-infant bonding | Definition: NICU’s guidance on building emotional attachment between parents and preterm infants.  Assessment standard: Includes instruction on kangaroo care, guided practice of skin-to-skin contact, and education on responsive interaction techniques to support parent-infant bonding. |
|  | 2.6.2 Emotional support for family members | Definition: NICU’s provision of psychological support and resources for families of preterm infants.  Assessment standard: Offers counseling, peer support access, and stress management strategies, family confirms understanding of available support. |
|  | 2.6.3 Assessment of maternal mental health | Definition: NICU’s systematic screening and comprehensive evaluation of maternal psychological well-being during preterm infant hospitalization.  Assessment standard: Uses validated tools (e.g., EPDS) to identify anxiety/depression.[1] |
|  | 2.6.4 Responsive parenting | Definition: NICU’s education on recognizing and responding to preterm infant behavioral cues.  Assessment standard: Covers cue identification (e.g., hunger, fatigue) and responsive interaction. |
|  | 3.1.1 Preparation of items: car seat, thermometer, infant clothing, diapers, formula, bottles (including specialized bottles), breast pump, and others | Definition: NICU’s guidance on preparing essential home care supplies for preterm infant discharge.  Assessment standard:   1. Confirms all listed items are available and appropriate; 2. family demonstrates correct use of 1-2 specialized items (e.g., specialized bottle, breast pump). [1] |
|  | 3.1.2 Family members can acquire essential home care skills: including bathing, diaper and clothing changes, medication administration, and umbilical cord care | Definition: NICU’s education on preterm infant daily care techniques.  Assessment standard: Covers bathing, diaper/clothing changes, medication administration, and umbilical cord care. |
|  | 3.1.3 Family members can master feeding skills: including feeding positions, techniques, and preparation | Definition: NICU’s training on preterm infant safe feeding practices.  Assessment standard: Covers feeding positions, formula preparation, and specialized bottle use. |
|  | 3.1.4 Family members can acquire skills in recognizing symptoms and signs: observing complexion and vital signs; monitoring preterm infant behavior; and assessing urination and defecation | Definition: NICU’s instruction on identifying preterm infant health cues and abnormal signs.  Assessment standard: Covers observation of complexion, vital signs, behavioral cues, and elimination patterns. |
|  | 3.1.5 Family members can identify emergencies and master first aid and safety skills: managing choking on milk and addressing respiratory pauses | Definition: NICU’s training on preterm infant choking and apnea first aid.  Assessment standard: Covers first aid for milk choking and respiratory pause stimulation, family demonstrates correct technique and prompt activation of emergency protocols. |
|  | 3.2.1 Temperature, humidity, and lighting are appropriately adjusted | Definition: NICU’s guidance on optimizing the home environmental conditions for preterm infants.  Assessment standard: Confirms temperature (22-24℃), humidity (50-60%), and lighting (soft, dim at night) are maintained within recommended ranges. [1,5] |
|  | 3.2.2 A safe and tranquil sleeping environment, such as a separate crib | Definition: NICU’s instruction on creating a safe sleep environment.  Assessment standard: Verifies use of a firm, separate crib with loose bedding; confirms no pillows, blankets, or toys are present in the sleep area. |
|  | 3.2.3 Smoke-free and free from any irritating odors | Definition: NICU’s education on maintaining a toxin-free home environment for preterm infants.  Assessment standard: Confirms the home is entirely smoke-free; identifies and eliminates sources of irritating odors (e.g., strong cleaning products, perfumes). |
|  | 3.2.4 Continuous supervision and companionship from family members around the clock | Definition: NICU’s guidance on ensuring constant monitoring and emotional support for preterm infants at home.  Assessment standard: Confirms a care schedule is in place to provide 24/7 supervision; at least one caregiver is present and attentive at all times. [1] |
|  | 3.3.1 The family possesses financial support resources | Definition: NICU’s assessment of the family’s financial capacity to sustain preterm infant care.  Assessment standard: Confirms access to health insurance, government subsidies, or community financial aid to cover medical and care expenses. |
|  | 3.3.2 The family has access to psychological support resources | Definition: NICU’s evaluation of the family’s access to mental health support.  Assessment standard: Confirms knowledge of counseling services, peer support groups, or mental health hotlines for caregiver well-being. |
|  | 3.3.3 The family are supported by social resources | Definition: NICU assessment of the family’s access to community and social support systems.  Assessment standard:   1. Confirms connection to at least one type of formal support resource (e.g., parent support organizations, community childcare assistance); 2. Documents the family’s ability to initiate contact with the resource provider independently (e.g., dial the service hotline, attend a support group meeting without NICU staff accompaniment). [1] |
|  | 3.3.4 At least two primary caregivers | Definition: NICU verification of a reliable caregiving team for preterm infant home care.  Assessment standard: Confirms at least two trained primary caregivers are available to share responsibilities and ensure continuous care. [1] |

**References**

1. The Neonatology Group of the Chinese Medical Association Pediatrics Society, the Editorial Committee of Chinese Journal of Neonatology, and the Neonatal Nursing Branch of the China Medical Education Association. Management on safe discharge for preterm infants (medical staff version): experts’ consensus[J]. Chinese Journal of Neonatology, 2022, **37(5)**: 385-394.
2. The Nutrition Committee of the Neonatology Physicians' Branch of the Chinese Medical Doctor Association, the Early-Infant Specialized Committee of the Neonatology Physicians' Branch of the Chinese Medical Doctor Association, the Editorial Committee of Current Pediatrics Journal, etc. Expert consensus on enteral nutrition management of preterm infants[J]. Current Pediatrics Journal, 2024, **26(6)**: 541-552.
3. Embleton ND, Jennifer Moltu S, Lapillonne A, et al. Enteral Nutrition in Preterm Infants (2022): A Position Paper From the ESPGHAN Committee on Nutrition and Invited Experts. *J Pediatr Gastroenterol Nutr*. 2023;**76(2)**:248-268.
4. American Academy of Pediatrics (AAP). Neonatal Care: A Compendium of AAP Clinical Practice Guidelines and Policies (2nd Edition). AAP Press, 2023.
5. Goodstein MH, Stewart DL, Keels EL, Moon RY; COMMITTEE ON FETUS AND NEWBORN, TASK FORCE ON SUDDEN INFANT DEATH SYNDROME. Transition to a Safe Home Sleep Environment for the NICU Patient. *Pediatrics*. 2021;**148(1)**:e2021052046.
